# Design and Synthesis of Low Surface Energy Coating with Functionalized Al_2_O_3_ Nanoparticles

**DOI:** 10.3390/ma16227223

**Published:** 2023-11-18

**Authors:** Siwei Pan, Yuanyuan Li, Yaohong Zhao, Qing Wang, Qing Hu, Yihua Qian, Chunqing He

**Affiliations:** 1 Electric Power Research Institute of Guangdong Power Grid Co., Ltd., Guangzhou 510080, China; kennyaoo@126.com (Y.Z.); sdytwangqing@163.com (Q.W.); 13926035192@163.com (Y.Q.); 2 School of Physics and Technology, Wuhan University, Wuhan 430072, China; yanttely@whu.edu.cn (Y.L.); 2020202020067@whu.edu.cn (Q.H.); hecq@whu.edu.cn (C.H.)

**Keywords:** Al_2_O_3_, low surface energy, optimization, nanocomposite coating

## Abstract

In a high-moisture environment where dust and coastal saltwater are prevalent, the stability of power equipment can be adversely affected. This issue can result in equipment downtime, particularly for transformers, severely disrupting the continuous operation of DC transmission systems. To address this challenge, a superhydrophobic modified fluorosilicone coating was developed, incorporating anti-stain properties. To tackle this issue comprehensively, an orthogonal experiment was conducted, involving six factors and three levels. The study focused particularly on assessing the impact of water-repellent recovery agents, nanofillers, antistatic agents, anti-mold agents, leveling agents, as well as wetting and dispersing agents on the coating’s surface tension. The results demonstrate that selecting an appropriate base resin and incorporating well-matched functional additives played a central role in effectively reducing the surface tension of the coating. Consequently, optimized coatings exhibited exceptional resistance to stains and displayed strong corrosion resistance.

## 1. Introduction

Fluoropolymers have found extensive application in functional organic superhydrophobic coatings due to their exceptional attributes, including remarkable thermal oxidation stability, resistance to harsh weather conditions, imperviousness to solvents, electrical insulating properties, and exceptional water and oil repellency, along with effective anti-fouling characteristics [1,2,3]. These materials exhibit low surface energy primarily due to the small size of fluorine atoms, which efficiently shield fluorocarbon atoms without causing any spatial resistance. Furthermore, the carbon–fluorine bonds resist polarization, resulting in diminished intermolecular forces and, consequently, lower surface tension [4,5]. Some reports even indicate that the surface tension of fluoropolymers can be as low as 6 mN/m [6]. Consequently, the synthesis of low-energy fluoropolymers and the development of superhydrophobic coatings or coatings based on them have garnered significant attention in recent years.

Silicone resins possess outstanding thermal oxidation stability, cold resistance, resilience against adverse weather conditions, water repellency, electrical insulation, and anti-fouling properties [7,8]. Recent research has focused on incorporating silicone polymers into fluoroacrylate copolymers to create fluorosilicone block copolymers [9,10,11]. However, fluorosilicone exhibits suboptimal adhesion to substrates due to its limited polarity. Typically, organic resins are employed to modify fluorosilicone, combining the excellent attributes of fluorosilicone resin with those of other organic resins, thus broadening its range of applications [12,13].

The amalgamation of fluorosilicone with polyester polymers enhances the overall properties of the resultant material. Polyester resin offers high strength and corrosion resistance [14,15], imparting a synergistic effect that reinforces and toughens the hybrid material. Moreover, the high cross-linking density and favorable compatibility of the blended polymer contribute to the uniformity of the film’s chemical and physical structure, preventing the buildup of stains and enhancing interfacial adhesion [16]. To achieve this goal, various additives can be employed. Notably, polysiloxane additives are known to reduce surface energy by facilitating the migration of polysiloxanes to the surface, with their structure primarily featuring -(CH_3_)_2_-SiO- groups [17,18]. The chemical structure, side chains, and end groups of these additives play pivotal roles in determining their properties and their impact on film performance. While conventional silicone additives have been regarded as versatile agents for many years, their limited effectiveness under outdoor conditions has led to the emergence of various functional additives that form strong covalent bonds with the coating matrix [19,20].

In this study, an orthogonal test design method was employed to investigate multiple factors and levels. This approach involves selecting a representative set of data points from a full-scale experiment, ensuring orthogonality. Specifically, three different fluorosilicones and five types of polyester were blended to assess their compatibility, adhesion, and surface tension in a hybrid clearcoat. Subsequently, an orthogonal test was conducted to compare the effects of six distinct functional additives on the surface tension of the fluorosilicone/polyester base clear coat. The functional nanofiller of aluminum oxide (Al_2_O_3_) was modified through the sol-gel method. Silane, as an interface compatibilizer, can significantly enhance the dispersion of alumina within the fluorosilicone coating, thereby further enhancing the mechanical stability of the coating.

## 2. Materials and Methods

### 2.1. Experimental Materials

Fluorosilicone resins (HLR-Si) were supplied by Dongfu Chemical Technology Co., Ltd. (Shanghai, China). The other two fluorosilicone resins (AG-2AF, AG-109F) were purchased from Sanaifu New Materials Co., Ltd. (Shanghai, China). Polyester resins (1265XL, YP2224) were supplied by Dongsheng Chemical Technology Co., Ltd. (Shanghai, China). The other three resins (FS-2970B, FS-4660, and FS-4470) were provided by Elementis Dekian Chemical Co., Ltd. (Shanghai, China). Propylene glycol methyl ether acetate (PMA), butyl acetate, dimethyl benzene, and ethylene glycol butyl ether were supplied by Tianjin Bodi Chemical Co., Ltd. (Tianjin, China). Curing agents (BAYER N3390, TPA-90SB) were supplied by Bayer AG and AsahiKASEI Co., Ltd. Water-repellent recovery reagent (TEGO-5001) was purchased from Deco Germany Co., Ltd. (Essen, Germany) Nanofiller (Al_2_O_3_) was supplied by Shanghai Macklin Biochemical Co., Ltd. (Shanghai, China). Antistatic reagent (BODEA) was supplied by Beijing Huaweiruike Chemical Co., Ltd. (Beijing, China). Anti-mold and anti-algae reagent (Copper pyrithione) was supplied by Shanghai Yuanye Biotechnology Co., Ltd. (Shanghai, China). Levelling reagent (DEQIAN-835) was supplied by Elementis Dekian Chemical Co., Ltd. Wetting and dispersing reagent (BYK-P 104S) was supplied by BYK-Chemie Co., Ltd. Formamide, diiodomethane, and xylene were purchased from Sinopharm Chemical Reagent Co., Ltd. All reagents were of analytical reagent and used without further purification. Distilled water was made in a laboratory.

### 2.2. Surface Modification of Al_2_O_3_

The process of surface modification of Al_2_O_3_ nanoparticles was conducted through a sol-gel chemical functionalization. This involved the in situ hydrolysis of two key compounds, namely ethyl orthosilicate (also known as Tetraethoxysilane, TEOS) and aminopropyltriethoxysilane (APTES). Initially, a specific amount of Al_2_O_3_ was dispersed into a solution consisting of TEOS and APTES in a ratio of 7:3 by weight. This mixture was mechanically stirred for 1 h and subsequently subjected to ultrasonication for 2 h. The pH was adjusted to 4.5 using glacial acetic acid, and the resulting mixture was allowed to sit for 48 h. Following this, a solution of NaOH with a mass fraction of 2.5% was utilized to raise the pH to 8.5, and gelation was initiated under water bath conditions at 65 °C. The Al_2_O_3_ was finally centrifuged and washed several times with a mixture of alcohol and water after the water bath. The resulting product functional Al_2_O_3_ was dried in freeze vacuum for further characterization.

### 2.3. Preparation of Coating

#### 2.3.1. Screening of Resin

The combination of three fluorosilicone resins (HLR-Si, AG-2AF, and AG-109F) and five polyester resins (1265XL, YP2224, FS-2970B, FS-4660, and FS-4470) was listed as one by one to measure the surface tension after film formation. The mass ratios of polyester resin and fluorosilicone resin (W_polyester_:W_fluoro_) were 1:11, 1.5:11, 2:11, 2.5:11, and 3:11. Polyester resin (1 g) and fluorosilicone resins (11 g) were dispersed in a mixed solution of PMA (19 g), butyl acetate (15 g), and xylene (1.5 g) by stirring for 30 min. Afterward, a 0.62 g curing agent was added to the mixture and stirred for a certain time. The resulting solution was coated on a clean glass slide and dried at 333 K for 7 h. All resins were prepared according to the mass ratios and methods mentioned above. The synthesis of fluorosilicone/polyester resins was performed following the same procedure reported above.

#### 2.3.2. Sample Preparation

The coating sample was based on fluorosilicone/polyester resins. Then, the additives were known as surface active reagents enabling the reduction in the surface tension. In this work, additives including water-repellent recovery reagent, nanofiller, antistatic reagent, anti-mold and anti-algae reagent, levelling reagent, and wetting and dispersing reagent were added to the resin mixture at various concentrations. These six additives chosen are listed in Table 1, whose chemical structures are differently functionalized and the effects have been reported [21].

According to the orthogonal, an orthogonal test is a method to analyze factors and levels of the representative experiment. In this study, the orthogonal experiment of six factors and three levels was used to fix the amount of film-forming resin and reaction conditions and change the concentration of additives, namely, the six factors are, respectively, the amount of water-repellent recovery reagent, nanofiller, antistatic reagent, anti-mold and anti-algae reagent, levelling reagent, and wetting and dispersing reagent. Each factor has three levels, and each level is shown in Table 1. The orthogonal experiment scheme is shown in Table 2.

The first step for coating was to synthesize additives ultrasonically dissolved in butyl acetate/PMA solution together and stirred at 600 r/min for 30 min. The mass ratio of butyl acetate: PMA was 1:1.5. Then, fluorosilicone resin and polyester resin were successively added into the mixed solution and stirred for 30 min. Afterward, 0.5 wt% curing agent was slowly dispersed in the mixture and stirred for 20 min. Finally, the finished product was filtered through an 80-mesh net. The resulting product was applied to a clean glass slide, and the obtained specimen was placed in an oven at 333 K for 10 h.

### 2.4. Testing and Characterization

The surface wettability, assessed through contact angle (CA), was determined using a contact angle goniometer (SL200KB, KINO Industry Co., Ltd., Boston, MA, USA). For each sample, the average value of measurements is obtained from three different locations.

The contact angle method of measuring the surface tension of material is based on the development of Young’s equation and is, by far, the most common way.

$$\gamma_{s}=\gamma_{s1}+\gamma_{1}\cos\theta$$

Van Oss et al. [22] suggested that the surface tension consists of the Lifshitz–van der Waals component *γ^LW^* and the Lewis acid–base component *γ^AB^*:

$$\gamma =\gamma^{LW}+\gamma^{AB}$$

Here, $\gamma^{AB}$ is separated into the acid parameter ($\gamma^{+}$) and base parameter ($\gamma^{-}$). It can be expressed as:

$$\gamma^{AB}=2{\left(\gamma^{+}\gamma^{-}\right)}^{1/2}$$

The test liquids would be at least one apolar and at least two polar, whose surface tension components $\gamma^{LW}$, $\gamma^{+}$, and $\gamma^{-}$ are known as listed in Table 3. The relation between the contact angle θ of test liquids and the solid surface tension is as follows:

$$\gamma_{1}\left(1+\cos\theta \right)-2{\left(\gamma_{S}^{\mathrm{LW}}\gamma_{1}^{\mathrm{LW}}\right)}^{1/2}=2{\left(\gamma_{S}^{+}\gamma_{1}^{-}\right)}^{1/2}+2{\left(\gamma_{S}^{-}\gamma_{1}^{+}\right)}^{1/2}$$

Good [23] suggests measurement with suitable pairs, such as water/formamide and water/ethylene.

The morphologies of Al_2_O_3_ were characterized by scanning electron microscopy (SEM, Hitachi, Tokyo, Japan). The chemical bonding states of the functionalized Al_2_O_3_ were analyzed by Fourier-transformed infrared spectroscopy (FTIR, Thermo Fisher Nicolet, Waltham, MA, USA).

According to GB/T 9286-1998 Paints and varnisher-Cross cut test [24]—Part 7, the cutting tool was held so that it was perpendicular to the surface of the sample cutting tool with uniform force and using an appropriate spacing guide, forming a specified number of cuts on the coating with a uniform cutting rate. The samples were coated on the sample substrate and cut by hand. After the cutting was finished, the samples were carefully examined with a visual magnifier in a well-lit environment and the test surfaces were graded by comparing them with the diagram.

The chemical resistance of the coating was tested by immersing the samples in NaOH (pH~12) and HCl (pH~4) for 7 days.

To demonstrate the anti-stain performance of the fabricated surfaces, the coated glass was placed in a Petri dish with a tilting angle of 15°. Both surfaces were contaminated with graphite particles that acted as artificial dirt. Water droplets were then dripped onto both contaminated surfaces. The anti-stain capability was examined by assessing whether different liquids such as dye, ink, milk, Coca-Cola, and slurry could flow down the surface without leaving any stains.

## 3. Results and Discussion

### 3.1. Analysis of Surface Tension of Fluorosilicone/Polyester Resins

**Figure 1:**
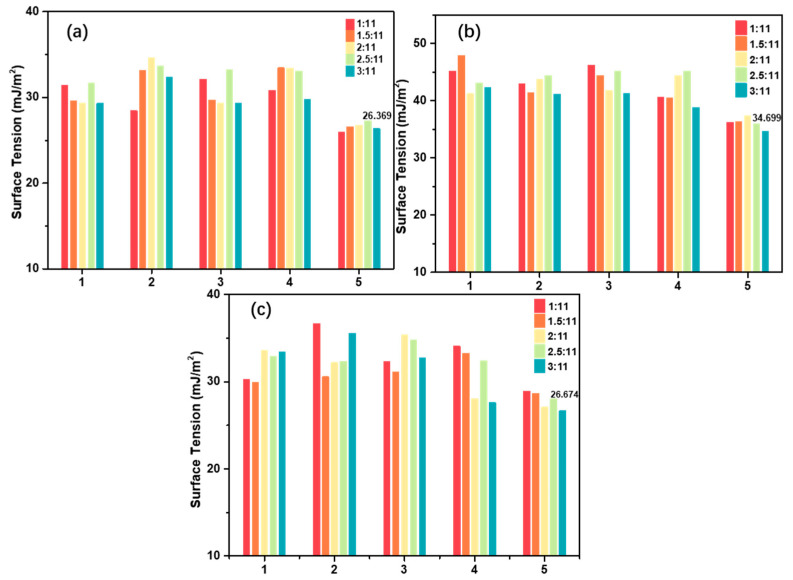
The surface tension of fluorosilicone/polyester resins without additives. (**a**) HLR-Si/polyester resins. (**b**) AG-2AF/polyester resins. (**c**) AG-109F/polyester resins. 1–5 stand for polyester resins 1265XL, YP2224, FS-2970B, FS-4660, and FS-4470, respectively.

The fluorosilicone/polyester resin coatings were investigated by measuring surface-free energy and assessing compatibility and adhesion. Figure 1 depicts the changes in surface tension concerning different resins.

As anticipated based on the wettability, the coatings exhibit a notably low surface energy (γ) owing to the presence of fluorine [25,26]. As illustrated in Figure 1, the inclusion of fluorosilicone/polyester resins indeed impacts surface tension, depending on the type of resin. The results indicate that the surface tension of AG-2AF/polyester resins (Figure 1b) ranges from 34.699 to 47.928 mJ/m^2^. Notably, HLR-Si/polyester resins (Figure 1a) display lower surface tension than AG-2AF and AG-109F. There exists an optimal ratio of fluorosilicone and polyester resins that results in reduced surface tension.

The compatibility of the resins and the adhesion of the clearcoat are presented in Table 4. It is evident from Table 4 that only certain combinations exhibit good compatibility, namely HLR-Si/FS-2970B, HLR-Si/FS-4660, HLR-Si/FS-4470, and AG-109F/FS-4660. Conversely, other pairings encounter compatibility issues, leading to the separation of resin phases. Consequently, the adhesion of the clearcoat is compromised due to the incomplete cross-linking of the matrix. According to GB/T 9286-1998, the first three levels are considered satisfactory for general use, with higher grades indicating better adhesion between the coating and substrate. Notably, HLR-Si/FS-4470 demonstrates superior adhesion capabilities. As a result, the HLR-Si/FS-4470 coating matrix enables lower surface tension with exceptional compatibility and adhesion.

### 3.2. Analysis of Surface Tension from Orthogonal Experiment Results

According to the results of the above discussion in Section 3.1, a clearcoat of HLR-Si/FS-4470 resins is selected for orthogonal design. The surface tension values of the coatings loaded with different concentrations of additives were measured. Table 5 shows the results of the surface tension of the coatings obtained by the orthogonal test. The various concentrations of additives resulted in the coating surface tension change. Results show that the surface tension reduction is more marked in sample 2. For further analysis, Table 6 shows the mean and range of the levels of each factor obtained from the calculations corresponding to Nos. 1–18 in Table 5. The range is the different value between the maximum and minimum of the mean value. The range of nanofiller is large, so the nanofiller has the most significant influence on the surface tension. The heightened hydrophobicity with greater nanofiller proportions can primarily be attributed to the increased surface roughness resulting from increased nanoparticles. According to the results, it can be seen that the factors affecting the surface tension of the coating are ranked B > D > E > A > F > C. The preferred formulation for the low surface tension coating is A2B3C3D2E2F2 (water-repellent recovery reagent 0.6 g, nanofiller 4.5 g, antistatic reagent 3 g, anti-mold and anti-algae reagent 0.6 g, levelling reagent 0.6 g, and wetting and dispersing reagent 0.6 g). The surface tension of the optimal sample A2B3C3D2E2F2 is 10.400 mJ·m^−2^. This is attributed to the incorporation of a fluorosilicone resin possessing low surface energy characteristics and a precise blend of six additives.

### 3.3. Analysis of Surface Characteristics of Low Surface Energy Coating

Based on the surface energy results obtained from orthogonal experiments, we conducted a single-factor experimental analysis with constant 0.6 g water-repellent recovery reagent, 3 g antistatic reagent, 0.6 g anti-mold and anti-algae reagent, 0.6 g levelling reagent, and 0.6 g wetting and dispersing reagent and varying quantities of f-Al_2_O_3_ nanoparticles to obtain SEM images of fluorosilicone/polyester coatings with different nano-filler levels.

**Figure 2:**
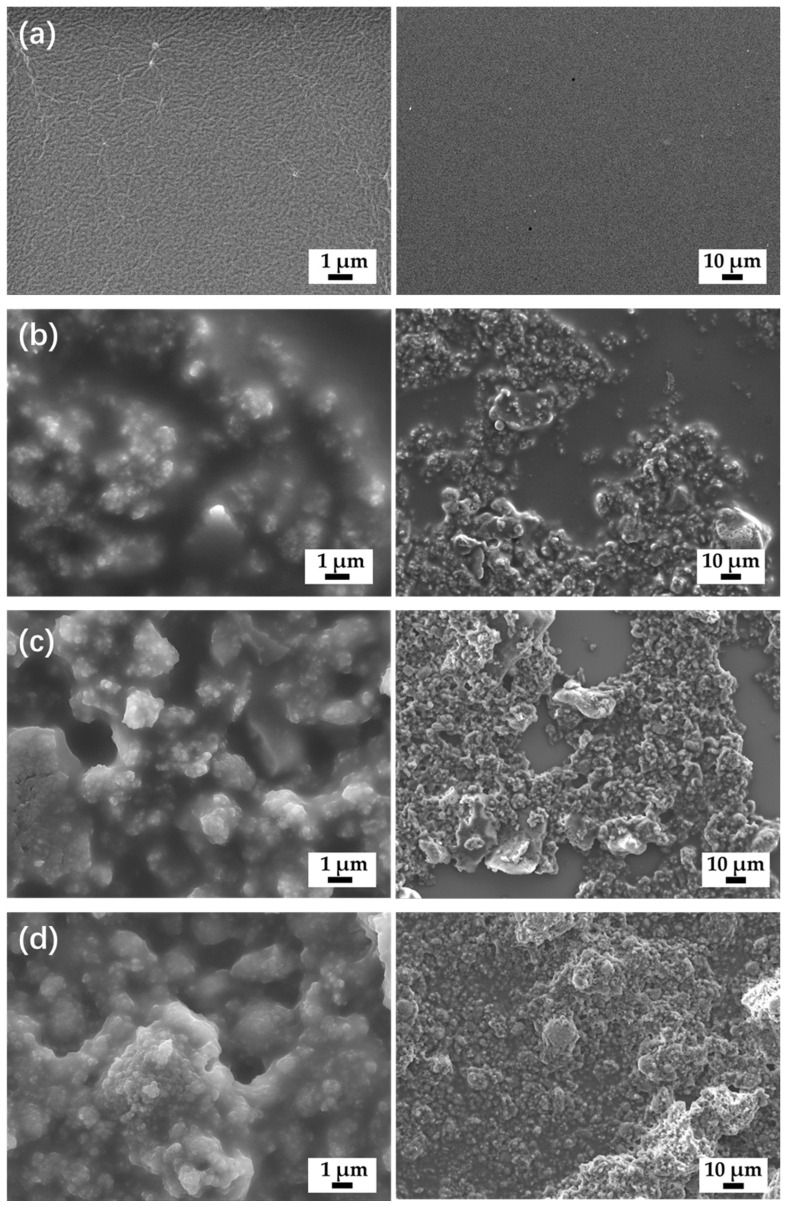
SEM images of fluorosilicone/polyester coating with nanofillers of different quantities of f-Al_2_O_3_ nanoparticles: (**a**) 0 g; (**b**) 1.5 g; (**c**) 3 g; (**d**) 4.5 g.

As shown in Figure 2a, the paint film without f-Al_2_O_3_ nanoparticles exhibited a smooth and uniform appearance. The introduction of f-Al_2_O_3_ nanoparticles led to an increase in coating surface roughness. With the addition of 1.5 g of f-Al_2_O_3_ (Figure 2a), the paint film contained fewer particles, resulting in a relatively even and structurally uniform surface. With a gradual increase in f-Al_2_O_3_, particle distribution on the paint film surface became more prominent. The surface morphology transitioned from relatively loose to denser. When f-Al_2_O_3_ addition reached 4.5 g, a layered micro-nanostructure was observed on the coating surface constructed by numerous cross-linked particles, which enhanced the air-trapping capability of the coating. However, excessive nanofiller significantly degraded both the surface quality and aesthetics of the paint film. Therefore, appropriate f-Al_2_O_3_ promoted surface roughness to form trapped air cushions at the gas–liquid–solid three-phase interface, which eventually decreases the surface energy of the coating.

**Figure 3:**
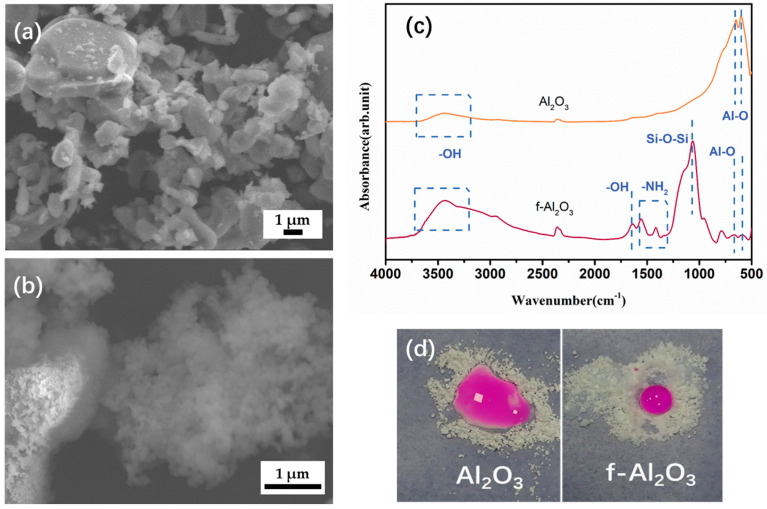
(**a**,**b**) SEM images, FTIR spectra (**c**), and (**d**) wettability comparison of Al_2_O_3_ and f-Al_2_O_3_ nanoparticles.

SEM analysis in Figure 3 reveals the morphological changes on the Al_2_O_3_ surface before and after modification. In Figure 3a, unmodified Al_2_O_3_ nanoparticles exhibit substantial aggregation due to van der Waals forces. As shown in Figure 3b, after surface modification, the swollen appearance and organic hydrolysis of the functionalized Al_2_O_3_ surface can be observed, both induced by the grafting of silane onto the alumina surface. This silane treatment, following sol-gel processing, effectively prevents aggregation and further improves the interface compatibility between Al_2_O_3_ and organic solvents.

FTIR analysis was conducted on Al_2_O_3_ and f-Al_2_O_3_ nanoparticles to assess their modification status, as shown in Figure 3c. The peaks appear at 595 and 656 cm^−1^, indicating the Al–O stretching mode, respectively [27]. A wide-ranging band near 3449 cm^−1^ is attributed to O–H stretching vibration [28]. Additionally, at 1650 cm^−1^, a band emerges as a result of the O–H bending of absorption of moisture [29]. Furthermore, a wide band within the 1200–1000 cm^−1^ range, along with a band at approximately 800 cm^−1^, can be attributed to the asymmetric and symmetric stretching vibrations of Si-O-Si bonds, respectively [30,31]. The observed peak at approximately ~2925 cm^−1^ may be attributed to the C-H stretching vibrations originating from the CH_3_ and CH_2_ groups present in the silane coupling agents [32]. Combining these results with the SEM images, it can be inferred that, after modification, some porous nanoscale organosilicon particles have coated the surface of Al_2_O_3_. Therefore, the presence of methyl and amino groups further confirms the successful grafting of APTES onto the surface of Al_2_O_3_.

In Figure 3d, the alterations in surface wettability between the pristine Al_2_O_3_ and the modified Al_2_O_3_ are depicted. As a comparison, hydrophobic modified Al_2_O_3_ exhibited non-wetting properties, confirming the hydrophobicity of f-Al_2_O_3_ nanoparticles. The exposed Al_2_O_3_ surface was relatively smooth with a low water contact angle value (71.2 ± 3.9°). In contrast, the silane-modified Al_2_O_3_ surface had some irregular particles with hydrophobicity and the contact angle value increased to 102.3 ± 2.0°.

**Figure 4:**
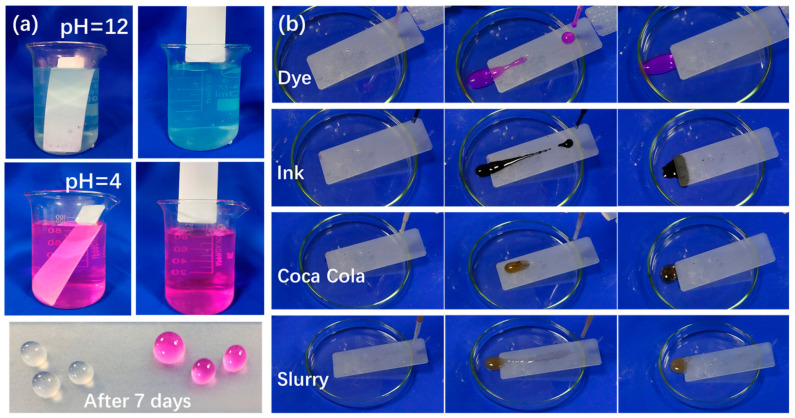
(**a**) Acid, alkali, and corrosive resistance test and (**b**) anti-stain test of the low-surface-energy coating.

Resistance to acid, alkali, and corrosion, as well as anti-stain properties, are additional requirements for protective coatings. In Figure 4a, for better observation, hydrochloric acid solution (pH = 4) and sodium hydroxide solution (pH = 12) were prepared with blue and pink dyes, respectively. The prepared low-surface-energy coatings were immersed in acidic and alkaline solutions for 7 days. The results indicate that, after one week of immersion, the surface remained in its previous state with no color adhesion. The droplets on the coating surface in both corrosive solutions maintained a spherical shape, indicating that the coating exhibited good long-term mechanical stability under corrosive conditions.

Figure 4b corroborates the anti-stain performance of the low-surface-energy coating against various stains. When dyes, ink, cola, and slurries were dropped onto the FS surface, the droplets maintained nearly spherical shapes on the surface, readily sliding apart without leaving any stains. The exceptional non-wettability of the coating is attributed to the presence of abundant air gaps between the modified nanoparticles on the coating’s surface and the liquid, coupled with lower surface energy, resulting in reduced adhesion to the surface.

## 4. Conclusions

In this study, we investigated surface tension, compatibility, and adhesion when blending fluorosilicone/polyester polymers with different resins. Notably, the compatibility of these two types of resins exhibited significant variations, directly impacting their interfacial adhesion performance. Among all the resins, HLR-Si/FS-4470 demonstrated superior overall properties. Furthermore, the functional additives within the HLR-Si/FS-4470 coating played a crucial role in determining its properties. Orthogonal experiments revealed that the nanofiller had the most significant impact on surface tension, followed by anti-mold and anti-algae reagent, levelling agent, water-repellent recovery reagent, wetting and dispersing reagent, and antistatic reagent. The optimal formulation for achieving a low-surface-tension coating included 0.6 g of water-repellent recovery reagent, 4.5 g of nanofiller, 3 g of antistatic reagent, 0.6 g of anti-mold and anti-algae reagent, 0.6 g of leveling reagent, and 0.6 g of wetting and dispersing reagent. Additionally, the composite coating exhibited a degree of resistance to staining. This research provides valuable insights into the development of low-surface-tension surfaces with hydrophobic characteristics and corrosion resistance.

## Figures and Tables

**Table 1:** Influencing factors and levels of orthogonal experiment.

Level	A	B	C	D	E	F
	Water-Repellent Recovery Reagent (g)	Nanofiller (g)	Antistatic Reagent (g)	Anti-Mold and Anti-Algae Reagent (g)	Levelling Reagent (g)	Wetting and Dispersing Reagent (g)
1	0.3	0.3	1	0.3	0.3	0.3
2	0.6	0.6	2	0.6	0.6	0.6
3	0.9	0.9	3	0.9	0.9	0.9

**Table 2:** Experimental design.

Sample No.	A	B	C	D	E	F
	Water-Repellent Recovery Reagent (g)	Nanofiller (g)	Antistatic Reagent (g)	Anti-Mold and Anti-Algae Reagent (g)	Levelling Reagent (g)	Wetting and Dispersing Reagent (g)
1	0.3	3	3	0.9	0.9	0.6
2	0.6	4.5	1	0.6	0.9	0.3
3	0.6	3	3	0.6	0.6	0.3
4	0.9	3	2	0.6	0.3	0.9
5	0.3	4.5	1	0.9	0.6	0.9
6	0.3	4.5	3	0.6	0.3	0.9
7	0.9	4.5	2	0.9	0.9	0.3
8	0.9	1.5	1	0.6	0.6	0.6
9	0.9	1.5	3	0.9	0.3	0.3
10	0.6	3	1	0.9	0.3	0.6
11	0.9	3	1	0.3	0.9	0.9
12	0.3	1.5	1	0.3	0.3	0.3
13	0.3	1.5	2	0.6	0.9	0.6
14	0.9	4.5	3	0.3	0.6	0.6
15	0.6	1.5	3	0.3	0.9	0.9
16	0.6	4.5	2	0.3	0.3	0.6
17	0.3	3	2	0.3	0.6	0.3
18	0.6	1.5	2	0.9	0.6	0.9

**Table 3:** Test liquids (mN/m).

Liquid	γ	$\gamma^{LW}$	$\gamma^{+}$	$\gamma^{-}$
Water	72.8	21.8	25.5	25.5
Formamide	58.0	39.0	2.28	39.6
Diiodomethane	50.8	50.8	≈0	≈0

**Table 4:** The compatibility and adhesion of coatings without additives.

Sample	Compatibility	Adhesion (Grade)
HLR-Si/1265XL	stratified	4
HLR-Si/YP2224	stratified	4
HLR-Si/FS-2970B	compatible	3
HLR-Si/FS-4660	compatible	2
HLR-Si/FS-4470	compatible	1
AG-2AF/1265XL	stratified	4
AG-2AF/YP2224	stratified	4
AG-2AF/FS-2970B	stratified	4
AG-2AF/FS-4660	stratified	3
AG-2AF/FS-4470	stratified	3
AG-109F/1265XL	stratified	4
AG-109F/YP2224	stratified	4
AG-109F/FS-2970B	stratified	3
AG-109F/FS-4660	compatible	3
AG-109F/FS-4470	stratified	3

**Table 5:** CA and surface tension.

Sample No.	CA (°)			Surface Tension(γ/mJ·m^−2^)
	Water	Formamide	Diiodomethane	
1	136	128	87	16.874
2	142	125	103	8.0873
3	135	126	101	9.484
4	133	117	83	16.165
5	140	125	95	10.774
6	140	127	97	10.009
7	141	128	92	12.128
8	129	119	88	15.704
9	130	118	90	13.881
10	135	125	94	12.396
11	134	121	92	12.479
12	128	117	80	20.045
13	142	128	98	9.528
14	140	127	97	10.009
15	133	115	83	16.312
16	140	129	94	11.868
17	133	123	90	14.504
18	127	114	89	14.13

**Table 6:** Range results of the surface tension in the orthogonal experiment.

Horizontal Mean	A	B	C	D	E	F
	Water-Repellent Recovery Reagent	Nanofiller	Antistatic Reagent	Anti-Mold and Anti-Algae Reagent	Levelling Reagent	Wetting and Dispersing Reagent
Mean 1	13.622	14.935	13.248	14.203	14.061	13.022
Mean 2	12.047	13.650	13.055	11.496	12.435	12.730
Mean 3	13.394	10.479	12.762	13.365	12.568	13.313
Range	1.575	4.455	0.486	2.707	1.625	0.583

## Data Availability

Raw data are available upon request.
